# Supplementary material for: Revealing the Roles of the JAZ Family in Defense Signaling and the Agarwood Formation Process in Aquilaria sinensis
Source: Int J Mol Sci. 2023 Jun 8;24(12):9872. doi: 10.3390/ijms24129872 (PMC10298640; doi:10.3390/ijms24129872)
Supplement: Supplementary file 1 [file ijms-24-09872-s001.zip › Supplementary Figures.pdf]

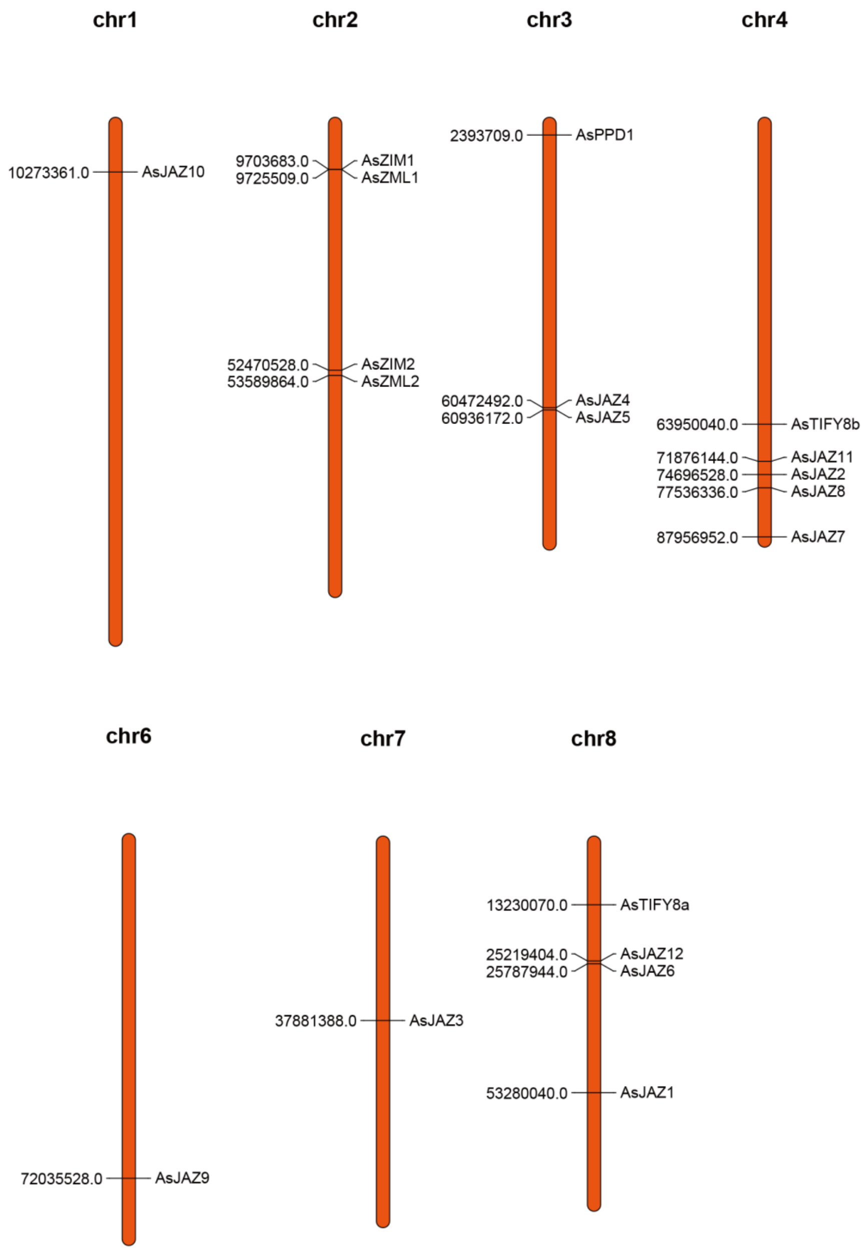

Figure S1. The distribution of the *AsTIFY* family genes on the chromosomes. ‘chr’ represents abbreviation of chromosome.

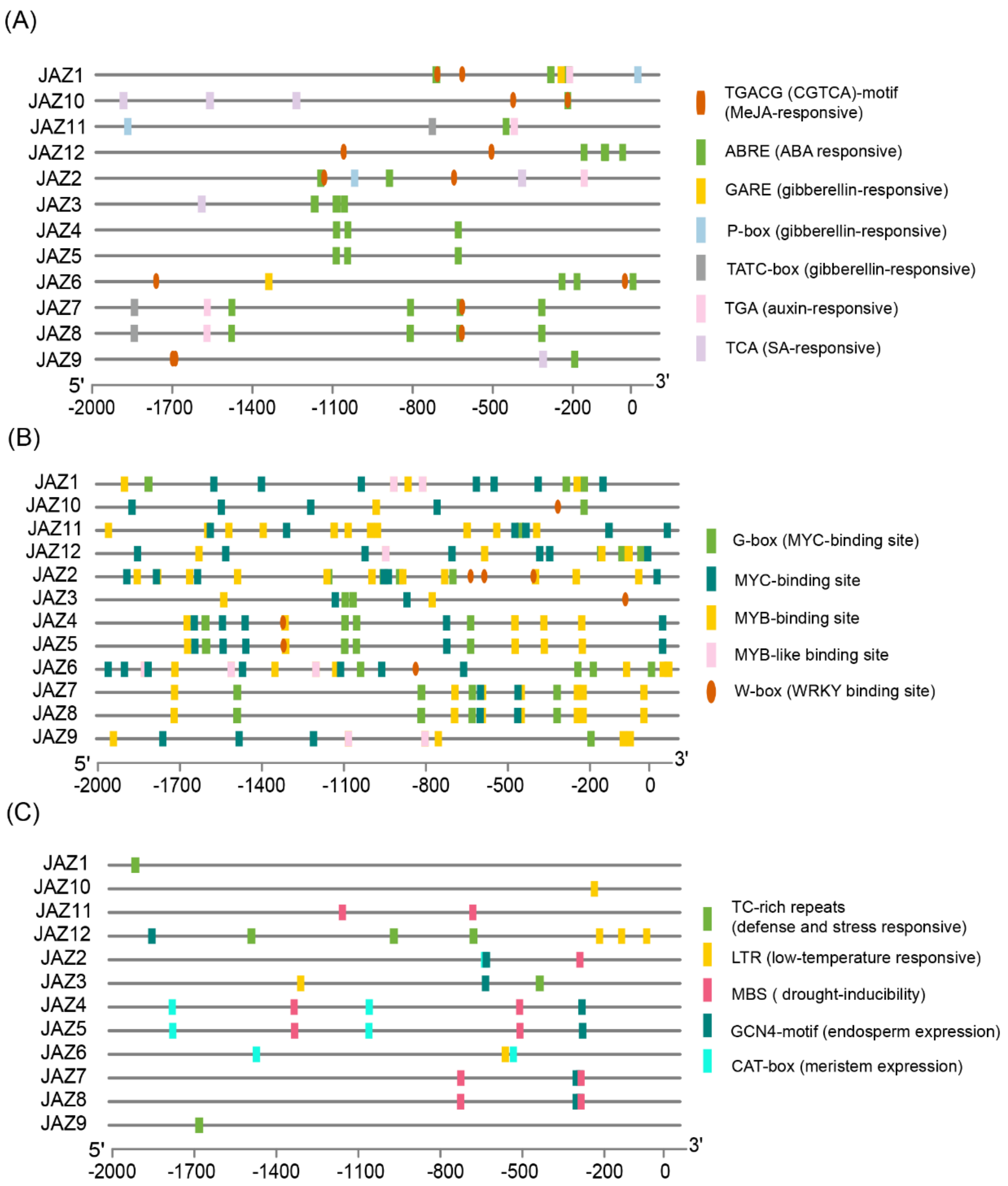

Figure S2. The putative *cis*-acting regulatory elements and motifs at the upstream 2kb of the translational initiation start sites of the *AsJAZ* genes. JAZ1~JAZ12 represent the *AsJAZ1~AsJAZ12* genes (**A**) Homologous hormone-responsive *cis*-acting elements. The TGACG (CGTCA)-motif are putative *cis*-acting elements involved in MeJA-responsiveness homologous with that in *Hordeum vulgare*. The ABRE (ABA responsive) element with sequence of "ACGTG (CACGT)", "CGCACGTGTC", "GACACGTACGT" or "GCCGCGTGGC" is homologous with ABA responsive elements in *Arabidopsis*, *Hordeum vulgare* or *Oryza sativa*. The GARE motif (TCTGTTG) is homologous with a putative GA-responsive element in *Brassica oleracea*, whereas the P-box (CCTTTTG) and TATC box (TATCCCA) are homologous with GA-responsive elements in *Oryza sativa*. The TGA (AACGAC) element is homologous with a putative auxin-responsive element in *Brassica oleracea*, whereas the TCA (CCATCTTTTT) element is homologous with a putative SA-responsive element in *Nicotiana tabacum*. (**B**) Possible transcription factor binding sites. W-box (TTGACC) represents a putative WRKY binding site

homologous with that in *Arabidopsis*; G-box represents the core sequence "C/TACGTG/T/C" that probably involved in light-responsive or MYC binding in various species; MYC (CATT/GTG or TCTCTTA) element is homologous with the putative *Arabidopsis* MYC binding sites; MYB (CAACA/TG, C/TAACCA, CCGTTG, TAACTG or AACCTAA) element is homologous with putative MYB-binding sites in various species; MYB-like sequence (TAACCA) is homologous with the putative binding sites of MYB-like transcription factors in *Arabidopsis*. (**C**) Possible tissue-specific and stress-related elements. TC-rich repeats (ATTCTCTAAC or GTTTTCTTAC) represents putative *cis*-acting elements involved in defense and stress responsiveness and homologous with that in *Nicotiana tabacum*; LTR (CCGAAA), a putative low-temperature responsive element homologous with that in *Hordeum vulgare*; MBS (CAACTG), a putative drought-inducing and MYB-binding site homologous with that in *Arabidopsis*; GCN4-motif (TGAGTCA), a putative endosperm-expression element homologous with that in *Oryza sativa*; CAT-box (GCCACT), a putative meristem-expression element homologous with that in *Arabidopsis*.

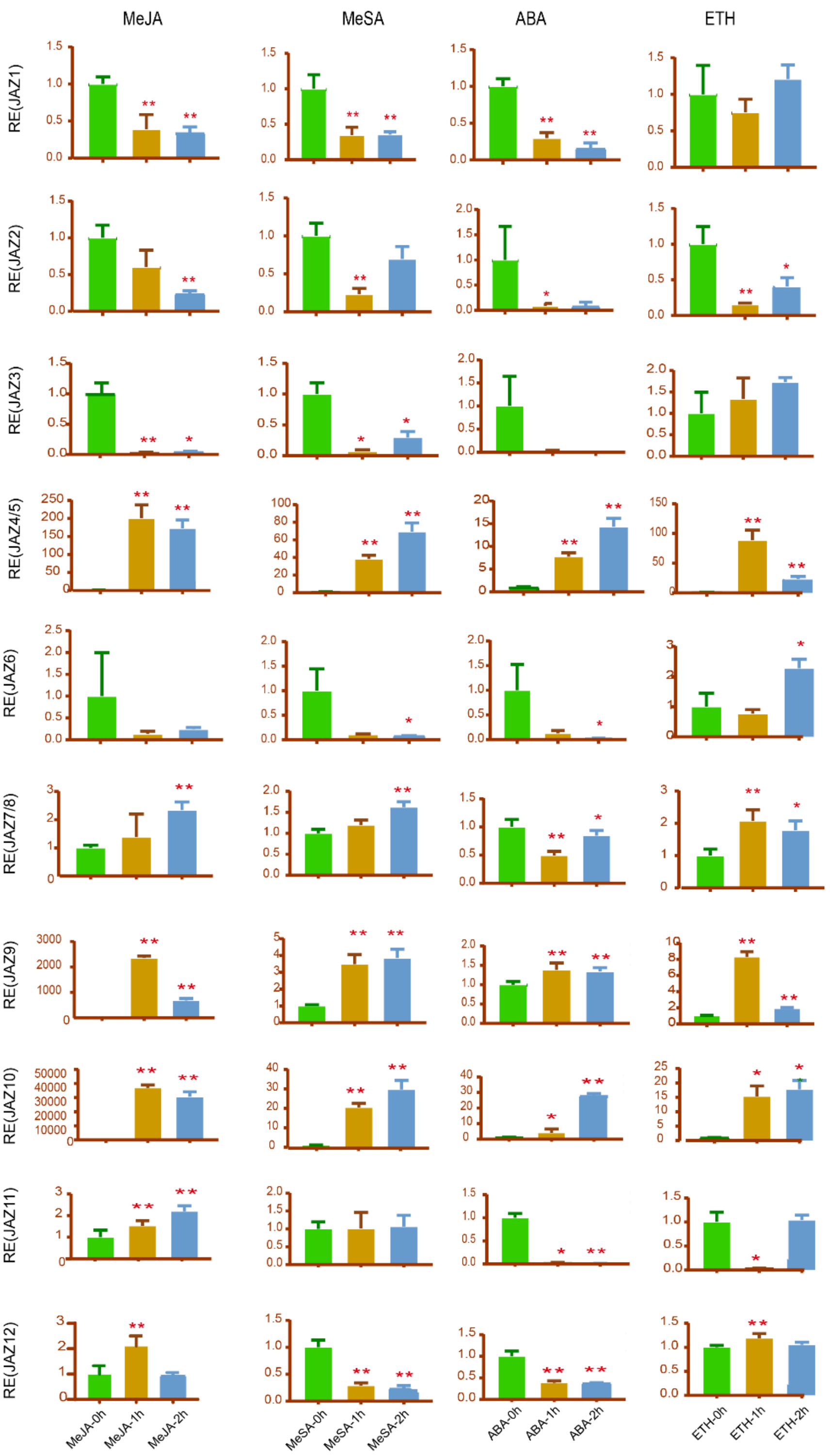

Figure S3. The relative expression changes of each *AsJAZ* gene under short-term treatments by various hormones. The expression data used are the same as Figure 5. The value at the y axis represent the relative expression levels of each individual *AsJAZ* gene and the expression level of each *AsJAZ* gene at Time 0 is arbitrarily set to 1. The significance of the expression difference between the control (0 h) and treatments (1 h or 2 h) for each gene was evaluated by Student's t-test, with "*" representing a significant correlation at the 0.05 (double-tailed) level and the "**" representing a highly significant correlation at the 0.01 (double-tailed) level.

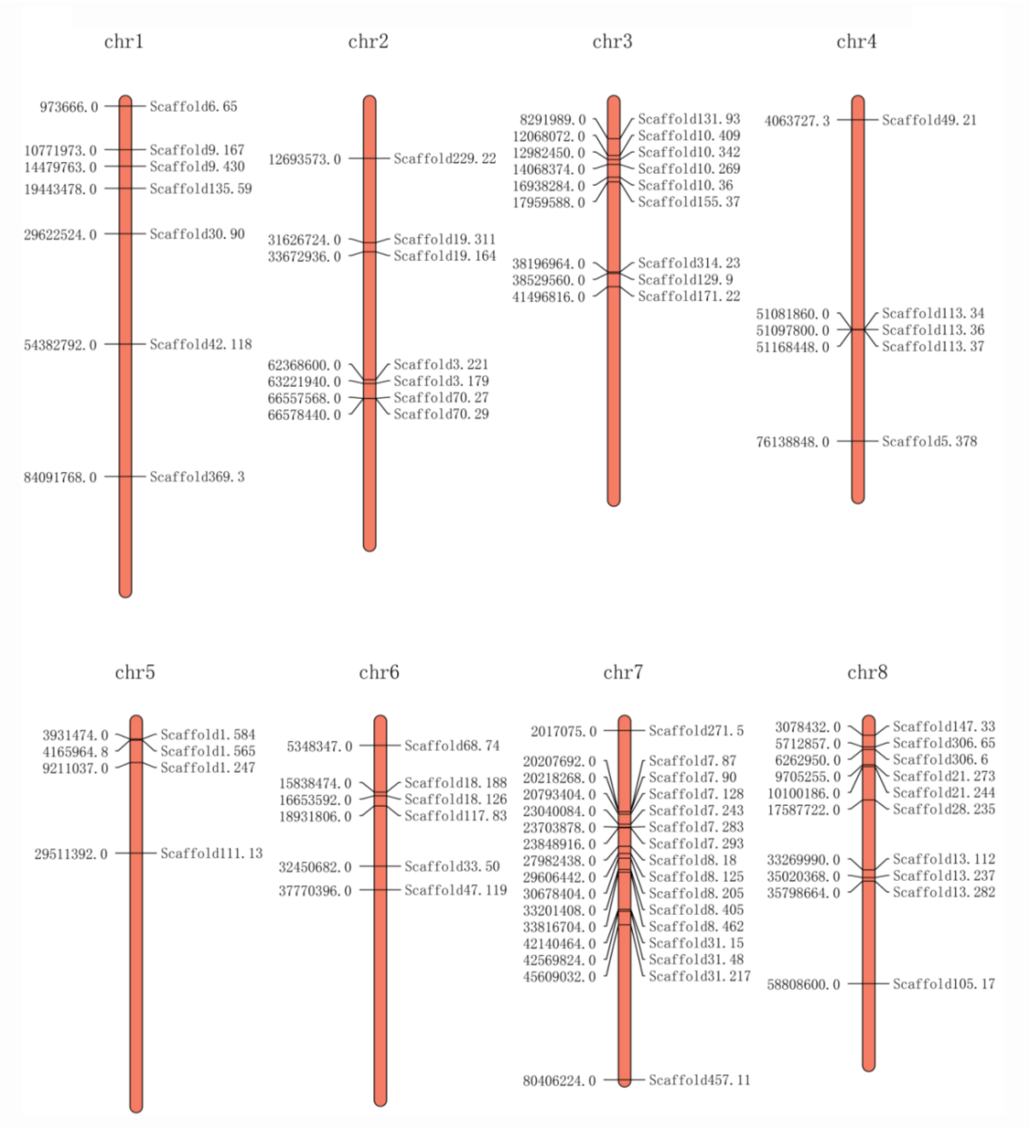

Figure S4. The distribution of the *AsWRKY* family genes on the chromosomes. The WRKY protein IDs are represented by the gene model ID (Scaffold number).
